# Addison’s disease in Italy: mortality and survival by etiology

**DOI:** 10.1007/s40618-026-02904-5

**Published:** 2026-05-22

**Authors:** Carlotta Keiko Vedolin, Chiara Sabbadin, Alessandro Mondin, Stefano Masiero, Mattia Barbot, Elisabetta Cavedon, Valentina Camozzi, Caterina Mian, Carla Scaroni, Fabio Presotto, Silvia Garelli, Carlo De Riva, Alessandro Delitala, Giorgio Radetti, Silvia Longhi, Carla Giordano, Valentina Guarnotta, Roberta Giordano, Antonella Meloni, Guido Di Dalmazi, Uberto Pagotto, Claudio Letizia, Giulia Marconcini, Fausto Bogazzi, Marco Cappa, Alessandra Fierabracci, Antonio Crinò, Anna Maria De Bellis, Mariluce Barrasso, Alberto Falorni, Valentina Morelli, Roberto Perniola, Francesca Pigliaru, Rosario Pivonello, Chiara Simeoli, Giuseppe Francia, Carolina Salerno, Donatella Capalbo, Giuseppe Picca, Antonio Stigliano, Malgorzata Gabriela Wasniewska, Filippo Ceccato, Corrado Betterle

**Affiliations:** 1 Department of Medicine-DIMED, University of Padova, Padova, Italy; 2 Endocrinology Unit, University Hospital of Padova, Via Ospedale Civile, 105, 35128 Padova, Italy; 3 Unit of Internal Medicine, Department of Medicine, Ospedale dell’Angelo, Mestre-Venezia, Italy; 4 Endocrine and Metabolic Diseases Unit, Department of Medicine, Ospedale dell’Angelo, Mestre-Venezia, Italy; 5 Medicina Interna I –Patologia Medica, Azienda Ospedaliera – Universitaria di Sassari, Sassari, Italy; 6 Marienklinik, Bolzano, Italy; 7 Department of Pediatrics, Hospital of Bolzano (SABES-ASDAA), Teaching Hospital of Paracelsus Medical University (PMU), Bolzano, Italy; 8 Unit of Endocrinology, Department of Health Promotion, Mother and Child Care, Internal Medicine and Medical Specialties, Università degli Studi di Palermo, Policlinico Paolo Giaccone, Palermo, Italy; 9 Division of Endocrinology, Diabetology and Metabolism, Department of Medical Sciences, Department of Biological and Clinical Sciences, University of Turin, Torino, Italy; 10 Clinica Pediatrica e Malattie Rare, Ospedale Pediatrico Microcitemico Antonio Cao, Cagliari, ASL Cagliari, Cagliari, Italy; 11 Division of Endocrinology and Diabetes Prevention and Care, IRCCS Azienda Ospedaliero-Universitaria di Bologna, Bologna, Italy; 12 Department of Medical and Surgical Sciences (DIMEC), Alma Mater Studiorum University of Bologna, Bologna, Italy; 13 Dipartimento di Scienze Cliniche Internistiche, Anestesiologiche e Cardiovascolari, Università Sapienza di Roma, Roma, Italy; 14 Dipartimento di Medicina Clinica e Sperimentale, Università di Pisa, Pisa, Italy; 15 Research Unit for Innovative Therapies in Endocrinopathies, Bambino Gesù, Children’s Hospital, IRCCS, Rome, Italy; 16 Bambino Gesù, Children’s Hospital, IRCCS Rome, Roma, Italy; 17 Life Sciences, Health and Health Professions Department, Link Campus University, Roma, Italy; 18 Center for Rare Diseases and Congenital Defects, Fondazione Policlinico Universitario A. Gemelli, IRCCS Rome, Roma, Italy; 19 Division of Endocrinology and Metabolic Diseases, University of Campania Luigi Vanvitelli, Napoli, Italy; 20 Department of Medicine and Surgery, University of Perugia, Perugia, Italy; 21 Endocrinology Department of Endocrine and Metabolic Diseases, IRCCS Istituto Auxologico Italiano, Milano, Italy; 22 Department of Pediatrics-Neonatal Intensive Care, University Hospital Vito Fazzi, Lecce, Italy; 23 Endocrinology Unit, University of Cagliari, Cagliari, Italy; 24 Dipartimento di Medicina Clinica e Chirurgia, Sezione di Endocrinologia, Diabetologia, Andrologia e Nutrizione Università Federico II di Napoli, Napoli, Italy; 25 Endocrinologia Policlinico GB Rossi di Verona, Verona, Italy; 26 Pediatric Endocrinology Unit, Department of Translational Medical Sciences, University Federico II, Napoli, Napoli, Italy; 27 Pediatric Endocrinology Unit, Department of Mother and Child, University of Naples Federico II, ENDO-ERN Center for Rare Endocrine Conditions, Napoli, Italy; 28 UOC Endocrinologia, Diabetologia e Malattie Metaboliche, Policlinico Riuniti Foggia, Foggia, Italy; 29 Department of Clinical and Molecular Medicine, Faculty of Medicine and Psycology, Sant’Andrea University Hospital, Sapienza University of Rome, Roma, Italy; 30 Department of Human Pathology of Adulthood and Childhood, University of Messina, Messina, Italy

**Keywords:** Addison’s Disease, Survival; Mortality, Autoimmune Polyendocrine Syndromes; Primary Adrenal Insufficiency, Genetic Addison’s Disease

## Abstract

**Background:**

Data on survival and mortality in Addison’s disease (AD) are contradictory: some studies suggest a mortality rate two times higher than in the general population, but this is not consistently reported in other studies.

**Methods:**

We evaluated survival and mortality in 1,826 Italian adult patients with AD of different etiologies, collected from 1947 to 2024 across 30 Endocrine Units. These outcomes were compared to those of the age- and sex-matched Italian reference population using a standardized mortality ratio (SMR) in a subgroup of 1,575 cases.

**Results:**

Sex and age adjusted survival analysis showed increased mortality in genetic AD, cancer-related-AD and cases with type 1 autoimmune polyendocrine syndrome (APS-1) compared to other etiologies. The SMR analysis demonstrated a significantly increased mortality risk of the overall patients with AD compared to the general population (SMR 1.34, *p* < 0.01). However, 4 etiologies presented survival similar to the general population: patients with type 2 or type 4 APS or isolated autoimmune AD (SMR 0.91, *p* = 0,57), vascular**/**post-tuberculosis AD (SMR 0.90, *p* = 0.69), bilateral adrenalectomy due to benign endocrine diseases (SMR 0.91. *p* = 0.64) and not defined AD (SMR 1.06, *p* = 0.93). These groups included 1,188 patients, representing 75.4% of the population. On the contrary, a significantly increased mortality risk compared to the general population was reported in the following 3 groups: patients with APS-1 (SMR 5.98, *p* < 0.01), genetic AD (SMR 5.09, *p* < 0.01), and cancer-related AD (SMR 4.64, *p* < 0.01), accounting for 387 patients (24.5%).

**Conclusion:**

Our study has shown that in adult patients with AD there is a large difference in survival and mortality based on the various etiological forms. In order to evaluate survival and mortality each form of AD must be considered separately and compared with an age- and sex-matched population.

## Introduction

Addison’s disease (AD), also called primary adrenal insufficiency, is a rare condition with a reported prevalence in Europe increasing from the 39 cases/million in the last century to 144/million on 2007 with an estimated incidence from 4.4 to 6.2 new cases/million/year [1]. In adults, the etiology of AD has varied over the decades: while in the first half of the twentieth century the main cause of AD was represented by the tuberculosis (TBC-AD), currently the most frequent cause is the autoimmune AD (AAD), which determines from 75 to 96% of cases in Europe, while the TBC-AD represents only 10–15% of forms. The remaining 5–10% of cases are due to other causes, such as infectious, vascular, genetic, surgical, neoplastic, and idiopathic [1]. In a study performed in Italy on 633 adult patients with AD, 77.7% were affected by AAD, 9% by TBC-AD, 4.6% by genetic forms (G-AD), 1.6% by cancer (C-AD), 1% by bilateral adrenalectomy (BA-AD), 0.6% by vascular forms (V-AD), 0.5% by other infectious, while in 5% the etiology was not defined (ND-AD) [2]. In children, the distribution of AD etiologies differs from that in adults, with a predominance of genetic causes and only approximately 10% of cases attributable to AAD [3].

Before 1940 AD was an invariably fatal condition due to absence of steroid replacement therapy, with a 1-year survival rate of about 20% or less. Most patients died within the first 5 years after diagnosis [4]. The discovery of cortisone in the late 1940 s dramatically improved the survival rate, with the exception of undiagnosed cases of AD or patients under poor social conditions [5].

Several studies have investigated the survival of patients with AD, with findings summarized in Table 1 [6–13]. The first study on the mortality in patients with AD was conducted in Sweden and published in 2006 [7]. It considered 1,675 adult patients, collected from 1987 to 2001, and demonstrated an increased mortality in subjects with AD compared to general population, especially among those with AD and not specified diabetes mellitus (DM). However, this study included only hospitalized patients and did not assess the different pathogenetic forms of AD. A subsequent Swedish study evaluated 3,299 hospitalized patients with “probable AAD” and reported a fourfold increase in mortality in patients with type 1 autoimmune polyendocrine syndrome (APS-1), and a twofold increase in those with type 2 autoimmune polyendocrine syndrome APS (APS-2) or isolated AAD (I-AAD) [8]. On the contrary, a subsequent Norwegian case-control study on 811 patients with AD did not confirm the previous data, reporting an overall normal mortality risk of patients with primary AD. Only patients diagnosed with AD before the age of 40 years showed a significantly elevated risk, likely corresponding to those affected by APS-1 [9]. In a subsequent nationwide observational cohort study cross-referencing the Swedish National Diabetes Register, the estimated Relative Risk (RR) of mortality in AD and DM group was increased about fourfold compared with controls with DM alone [11]. Another retrospective cohort study was done using the United Kingdom general practitioner database: 2,052 patients with AD not specified for etiology were evaluated for mortality by Hazard Ratio (HR) with matched controls. In AD the HR was increased twofold [13]. Moreover, three studies evaluated the mortality in patients with congenital adrenal hyperplasia (CAH), demonstrating a significant increased rate of mortality compared to the general population in United Kingdom [6, 12] and in Sweden [10] (see Table 1). All these data on mortality in patients with AD demonstrate considerable variability, likely due to differences in data collection methods, patient age, geographical provenance and underlying etiology of AD and are confirmed in a very recent systematic review and meta-analysis [14].

**Table 1:** Previous studies evaluating survival of patients with Addison's disease

Ref. *N*°	*N*° of Patients	Mean age Years	Period of follow-up	Risk Ratio (RR), Standardized mortality Ratio (SMR), Hazard Ratio (HR), Confidence Interval (CI).	Causes of death
[6]	333 CAH	ND	1964–1996; UK	Total SMR 3.4, CI 1.5–6.6	Adrenal crisis, often after infections
[7]	1,675 AD	52.8	1987–2001; Swedish hospitals	Total RR 2.19 CI 1.91–2.51 Men RR 1.8 CI 1.29–2.06 Women RR 2.86 CI 2.54–3.20 Men AD + DM RR 1.82 CI 1.29–2.06 Women AD + DM RR 1.52 CI 1.11–2.07	CV, cancer, endocrine, respiratory and infectious diseases
[8]	3,299 probable AAD	57	1964–2004; Swedish hospitals	Total SMR 2.7 CI 2.6–2.8 Men SMR 2.5 CI 2.3–2.7 Women SMR 2.9 CI 2.7–3.0.7.0 APS-1 SMR 4.6 CI 3.5–6.0.5.0 APS-2 SMR 2.1 CI 1.9–2.4 Isolated AAD 2.8 CI 2.6–2.9	CV, cancer, respiratory and endocrine disorders
[9]	811 AD	ND	1943-2005; Norwegian wide search of diagnosis registries	Total SMR 1.15 CI 0.95–1.34 Men SMR 1.10 CI 0.80–1.39 Women SMR 1.18 CI 0.92–1.44 Patients with AD < 40 yrs SMR 1.50 CI 1.09–2.01	Adrenal crisis, infections, and sudden death
[10]	588 CAH	ND	1910-2009; Swedish study Born	Men HR 2.3 CI 1.2–4.3 Women HR 3.5 CI 2.0–6.0	Adrenal crisis, CV and cancer
[11]	630 AD + DM	19.1	1996–2012; Swedish National Diabetes Register	Total AD + DM HR 3.89 CI 2.84–5.32 AD + DM-1 HR 4.28 CI2.59-6.95.95 AD + DM-2 HR 3.81-C2.52-5.75.75	CV, diabetic complications, infections, other unknown causes
[12]	270 CAH	ND	UK retrospective matched-cohort study	Total HR 5.17 CI 2.8–9.5 Men HR 6.81; Women HR 4.69	ND
[13]	2052 AD	ND	UK database	Total HR 1.83 CI 1.66–2.02	CV, cancer, respiratory diseases, infections, adrenal crisis

AAD, autoimmune Addison’s disease; AD, Addison’s disease; CAH, congenital adrenal hyperplasia; CV; cardiovascular; DM, diabetes mellitus; DM-1, type 1 diabetes mellitus; DM-2, type 2 diabetes mellitus; ND, not defined

The aim of the present study is to evaluate the survival and mortality in a population of Italian patients with AD considering the different etiologies (autoimmune and with other forms of AD) and comparing these data with those of the Italian reference population matched by age and gender.

## Materials and methods

### Patients

**Fig. 1:**
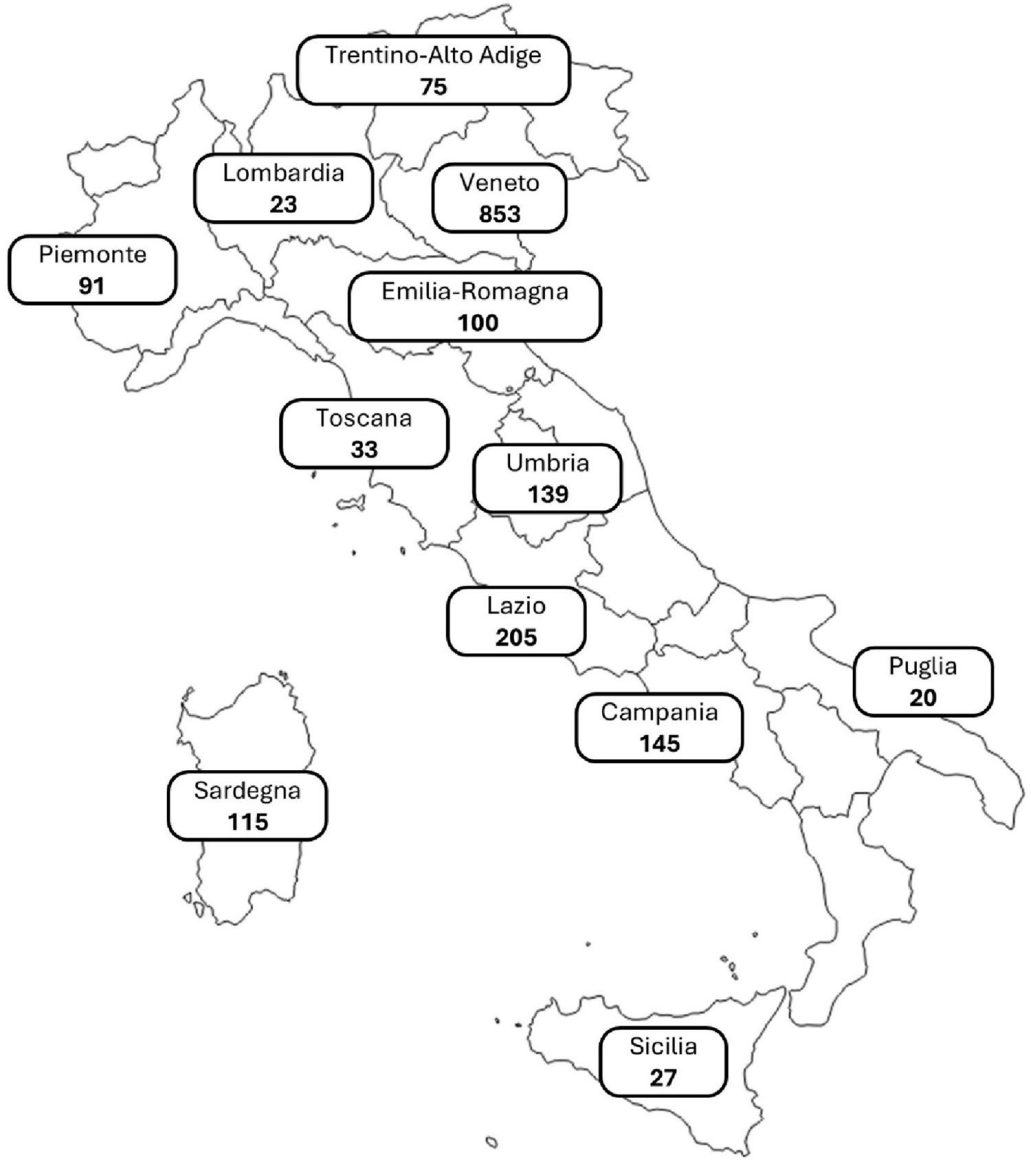
Number of patients collected from the centers based on the region of their reference Endocrine Unit

The study included 1,826 Italian adult patients with primary AD of various etiologies, collected from 1947 to 2024 across 30 Endocrine Units from 12 Italian regions (Fig. 1).

For each patient, we collected demographic data including date of birth, year of AD diagnosis, year of last follow-up or death, underlying etiology of AD, and the presence or absence of associated autoimmune diseases. In the 237 patients diagnosed between 1947 and 1962 prior to the discovery of adrenal cortex autoantibodies, the etiology of AD was identified based on clinical criteria: TBC-AD cases on the basis of the TBC history and by chest X-ray; APS-1 cases on the association of AD with hypoparathyroidism and/or candidiasis, APS-2 cases on the association of AD with hypothyroidism and/or DM-1. Cases with initially uncertain etiology tests were performed later during follow-up. When uncertainty persisted over time, patients were included in the ND-AD group. The study was performed according to the principles of the Helsinki declaration. The study was approved by the Ethical Committee of the University Hospital of Padua, Italy (N° 1373P and 1583P).

According to these data the patients were divided into two main groups:

**1) AAD**, which included 1,174 patients subdivided in two subgroups: the first affected by APS-1 and the second affected by type 2 or type 4 APS or I-AAD (APS-2/APS-4/I-AAD).

**2) Other forms of AD**, which included 652 patients subdivided in the following 5 subgroups: vascular or post-TBC (V/TBC-AD), G-AD, BA-AD due to benign endocrine diseases, C-AD and ND-AD.

### Statistical analysis

Categorical variables were reported as counts or percentages, and quantitative variables as median and interquartile ranges [IQR]. The comparisons between independent groups were performed with a chi squared test for categorical variables and with a Mann–Whitney sum rank test for quantitative variables. Survival analysis was performed using Kaplan–Meier analysis and Cox regression (under the assumption of constant hazard ratios over time); Cox regression model was then adjusted for sex and age at diagnosis. Sensitivity analyses were performed by censoring survival curves at various time points (i.e., every 10-years). In case of multiple comparisons, the Bonferroni correction was used; to ease readability the reported p values have been multiplied for the number of comparisons (adjusted p-value) to allow a direct assessment of significance against the conventional threshold of 0.05. We compared the mortality in our cohort to that of the general population by calculating the standardized mortality ratio (SMR) as described elsewhere [15]. Expected death rates were calculated from the yearly age- and sex-specific death rates provided by the Italian National Statistics Institute (ISTAT) database (2011–2022). From this reason the analysis of the initial sample, was reduced to a population of 1,575 patients for SMR analysis.

A SMR > 1 indicated increased mortality, whereas a SMR < 1 indicated reduced mortality. A Fisher exact test was carried out to assess significant differences with respect to the general population and calculating the 95% confidence interval (95% CI) for SMR. The threshold for statistical significance was set at p-value < 0.05. Statistical analyses were conducted in [16, 17]. An open-source calculator was also used to perform the Fisher exact test [18].

## Results

The cumulative observation period for patients with AD was 31,701 patient-years, with a median follow-up of 15 (6–26) years per patient. A total of 1,174 patients (64.3% of the total cohort) were diagnosed with AAD, while the remaining 652 (35.7% of the total cohort) had other forms of AD. Among those with AAD, 1,040 patients (56.9%) had APS-2/APS-4/I-AAD, whereas 134 patients (7.3%) had AAD as part of APS-1. Among the other forms of AD, 109 patients (6%) had V/TBC-AD, 202 (11.1%) G-AD, 86 (4.7%) BA-AD, 118 (6.5%) C-AD and 137 (7.5%) had ND-AD. The Table 2 summarizes female-to-male ratio, the median age at diagnosis of AD, the median duration of follow-up, the median age at last follow-up, number and proportion of deaths, and the median age at death across the different groups and subgroups.

**Table 2:**
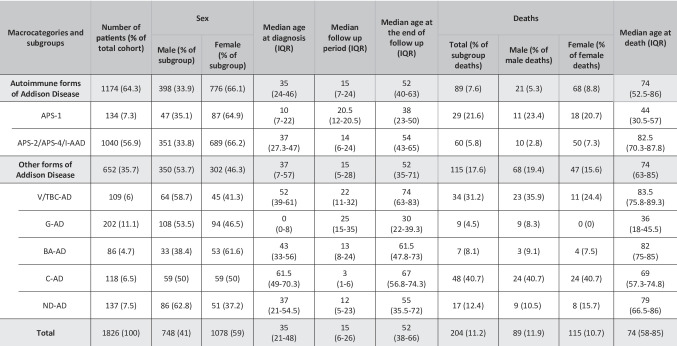
Epidemiological data of patients with AD (IQR= Inter-Quartile Range). Of note, a median age of 0 years indicates that most cases were diagnosed in the first year since birth, more precise information was unavailable

APS-1,type 1 autoimmune polyendocrine syndrome; APS-2/APS-4/I-AAD, type 2 autoimmune polyendocrine syndrome/type 4 autoimmune polyendocrine syndrome/isolated autoimmune Addison’s disease, BA-AD, bilateral adrenalectomy-related Addison’s disease; C-AD, cancer-related Addison’s disease; G-AD, genetic-related Addison’s disease, ND-AD, not defined Addison’s disease; V/TBC-AD, vascular- or post-tuberculosis-related Addison’s disease

The median age at diagnosis varied among the different forms of AD, as did the median duration of follow-up, except for certain disease groups. Notably, patients with C-AD consistently showed a significantly shorter median follow-up duration compared to all other subgroups, as expected from the underlying pathology.

During the follow-up, a total of 204 deaths were recorded (11.2%), with a median age at death of 74 years. The distribution of deaths and the median age at death differed between AAD and other forms of AD. In the AAD group, 89 deaths occurred among 1,174 patients (7.6% of the category), with a median age at death of 74 years. In contrast, in the non-autoimmune group, there were 115 deaths among 652 patients (17.6% of the category), with a median age at death of 64 years.

Among the AAD forms, the APS-1 group presented earlier and higher mortality compared to the other groups with AAD (total deaths 29 out of 134 cases, 21.6%, with a median age at death of 44 years vs. 60 out of 1,040 cases, 5.8%, with a median age at death of 82.5 years in the APS-2/APS-4/I-AAD group). Among the other forms of AD, the highest rate of deaths was recorded in the C-AD group (48 deaths out of 118 patients, 40.7%, with a median age at death of 69 years), followed by the V/TBC-AD (34 deaths out of 109 patients, 31.2%, with a median age at death of 83.5 years). Among the G-AD form, 8 out of 9 deaths (88.9%) interested patients affected by adrenoleukodystrophy, despite this disease accounting for only 14.3% of the category (other cases were due to CAH in 75.3% and other rare genetic causes in the remaining 10.4%).

Of the 204 deaths reported, the leading cause was neoplastic cachexia (63 cases, 30.1% of total deaths), followed by cardiovascular events (28 cases, 13.7%), infections (23 cases, 11.3%), adrenal crisis (5 cases, 2.5%), hepatorenal failure (5 cases, 2.5%), and accidental or intentional trauma (5 cases, 2.5%). In 75 cases (36.8%), the cause of death was either not reported or did not fall into the aforementioned categories. Causes of death for each etiology are reported in Table 3.

**Table 3:**
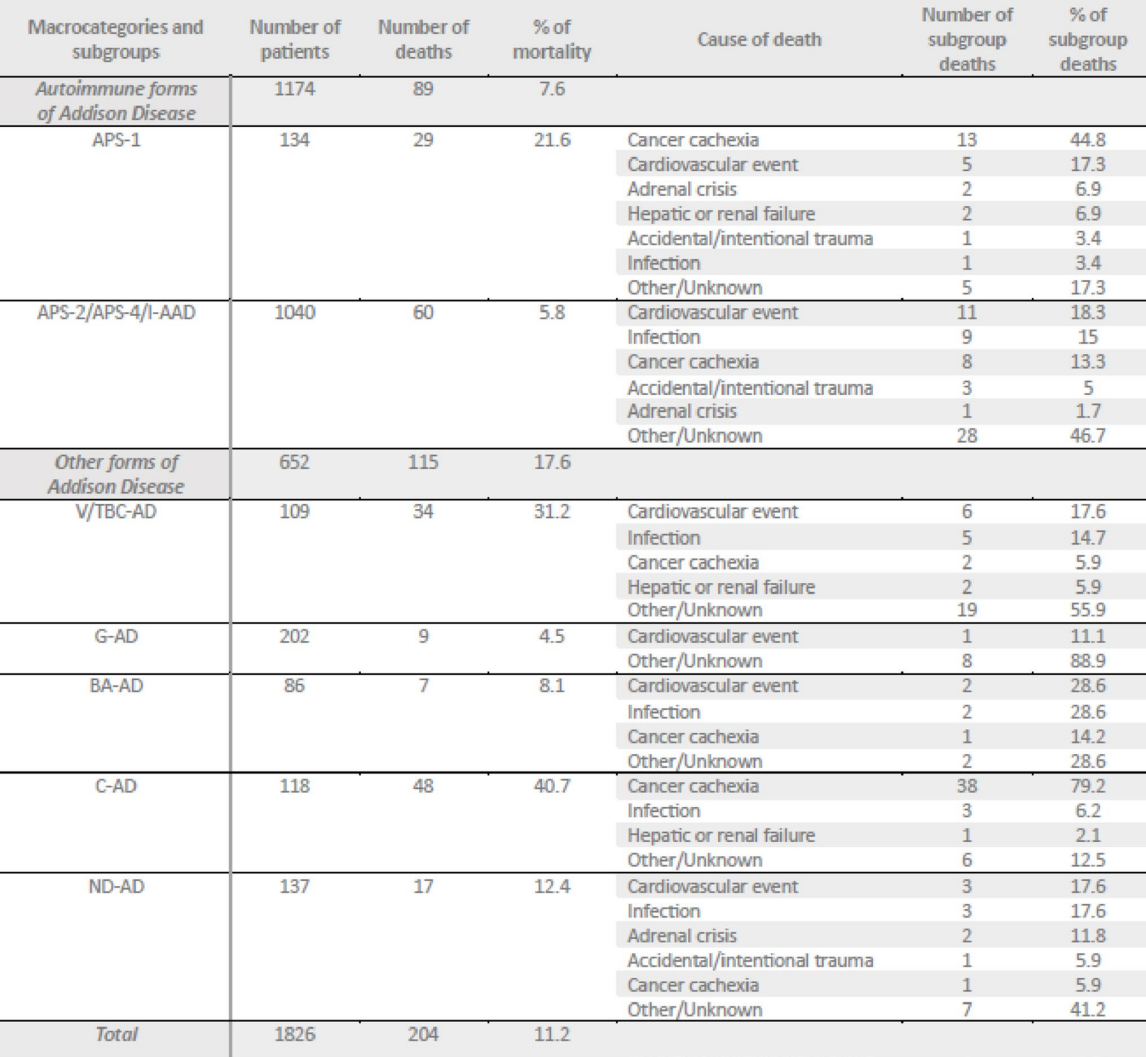
Different causes of deaths according to the various etiologies of Addison's disease

APS-1,type 1 autoimmune polyendocrine syndrome; APS-2/APS-4/I-AAD, type 2 autoimmune polyendocrine syndrome/type 4 autoimmune polyendocrine syndrome/isolated autoimmune Addison’s disease, BA-AD, bilateral adrenalectomy-related Addison’s disease; C-AD, cancer-related Addison’s disease; G-AD, genetic-related Addison’s disease, ND-AD, not defined Addison’s disease; V/TBC-AD, vascular- or post-tuberculosis-related Addison’s disease

### Survival analysis

**Fig. 2:**
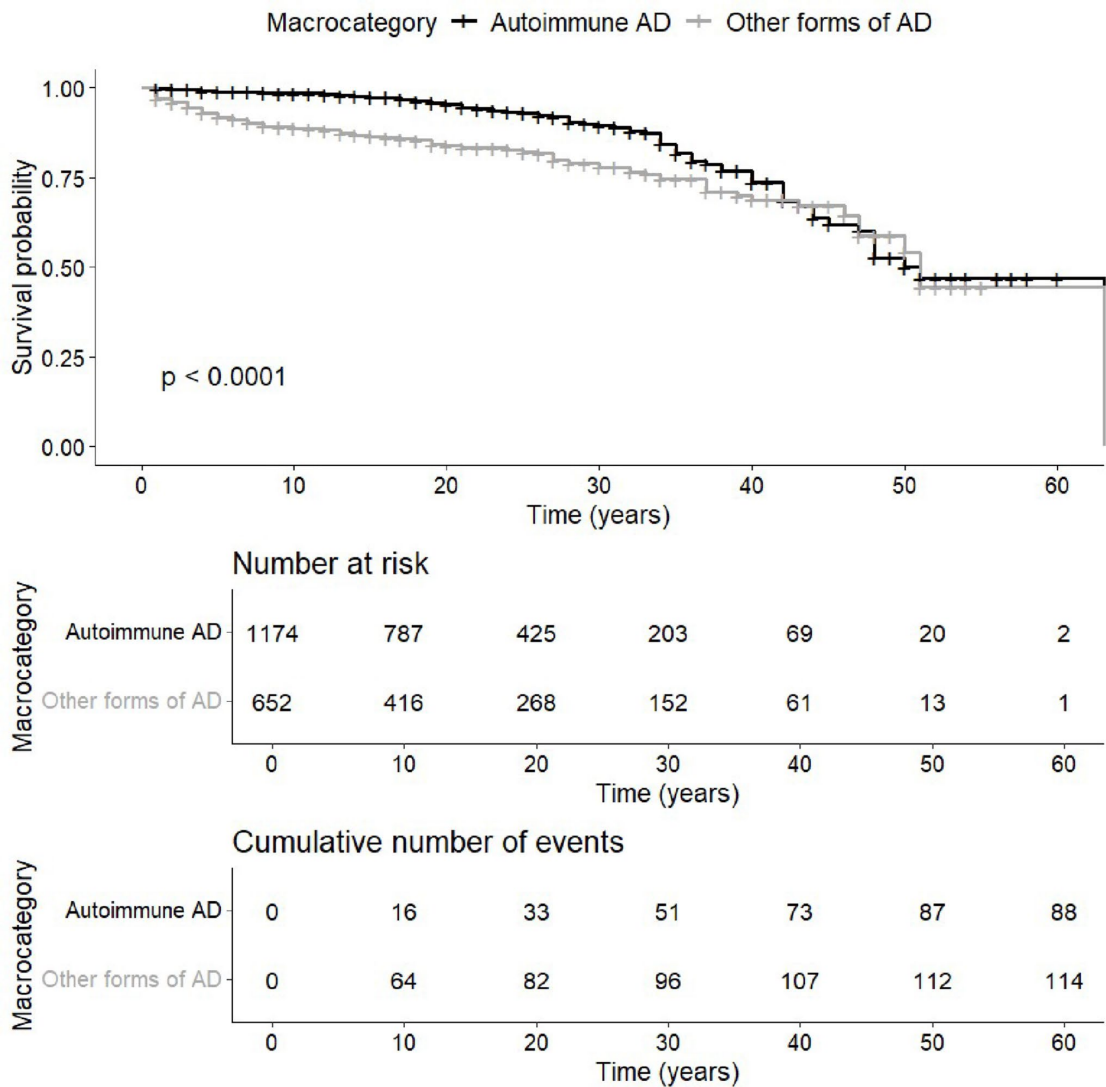
Kaplan–Meier survival curves comparing autoimmune AD and other forms of AD. Results were consistent at all censoring points (10, 20, 30, 40, 50 years). AD, Addison’s disease

Survival curve analysis of the two macro-categories (AAD and other forms of AD) revealed significantly lower survival in the other forms of AD compared to AAD forms (*p* < 0.0001), confirmed by sensitivity analyses censoring data at various time points (10, 20, 30, 40, 50 years of follow-up) (Fig. 2). When considering gender, a higher mortality of AAD cases still emerged in males (significant at all censoring points) and females (significant when censoring up to 40 years, a trend thereafter with *p* = 0.064).

**Fig. 3:**
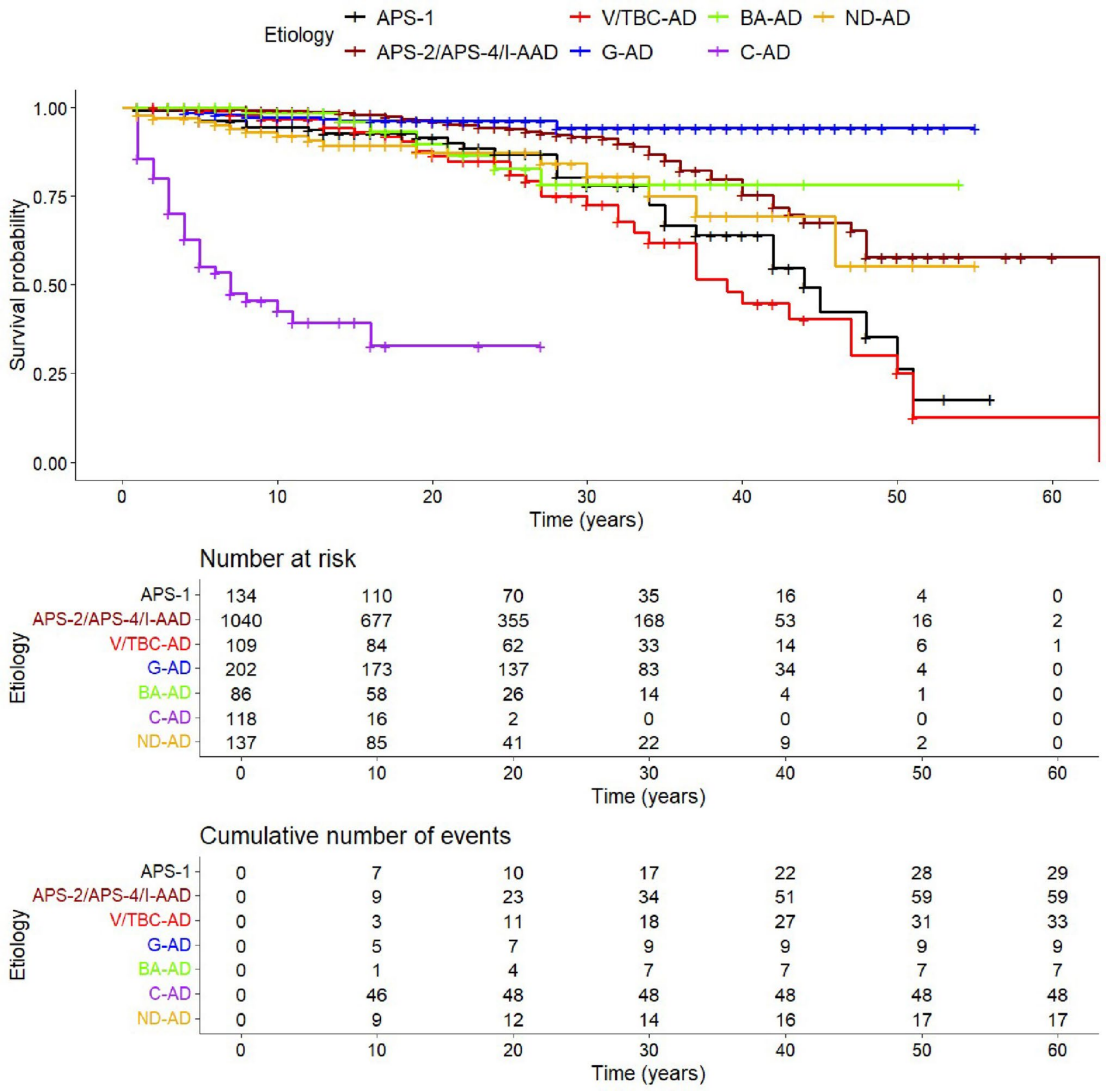
Kaplan Meier curves of survival based on the AD etiology. APS-1, type 1 autoimmune polyendocrine syndrome; APS-2/APS-4/I-AAD, type 2 autoimmune polyendocrine syndrome/type 4 autoimmune polyendocrine syndrome/isolated autoimmune Addison’s disease, BA-AD, bilateral adrenalectomy-related Addison’s disease; C-AD, cancer-related Addison’s disease; G-AD, genetic-related Addison’s disease, ND-AD, not defined Addison’s disease; V/TBC-AD, vascular- or post-tuberculosis-related Addison’s disease

Survival curves for the different etiological forms of AD are plotted in Fig. 3 and were compared pairwise using log-rank test and cox regression to assess potential differences in survival, applying Bonferroni correction and the above-described sensitivity analyses. C-AD showed significantly lower survival compared to all other etiological categories at all censoring times of follow-up (*p* < 0.01). Patients with APS-1 and V/TBC-AD also showed increased mortality compared to those with APS-2/APS-4/I-AAD (respectively HR 2.50, 95%CI [1.59; 3.85], *p* < 0.01 and HR 3.20, 95%CI [2.09; 4.90], *p* < 0.01) and G-AD (respectively HR 6.04, 95%CI [2.86; 12.76], *p* < 0.01 and HR 7.79, 95%CI [3.72; 16.31], *p* < 0.01); these trends were consistent when censoring at 30, 40 and 50 years of follow-up, but not in the earlier years. In addition, patients with ND-AD showed increased mortality compared to G-AD on the long term (starting from 30 years of censoring time, HR 3.98, 95%CI [1.72; 9.22], *p* < 0.03) and compared to APS-2/APS-4/I-AAD in early years (up to 40 years of censoring time, HR 2.49, 95%CI [1.42; 4.37], *p* = 0.03).

A Cox model accounting for sex and age at diagnosis was then created. Gender did not significantly impact mortality, whilst age at diagnosis was a significant predictor of mortality (HR 1.08 in uncensored model, 95%CI [1.07; 1.10], *p* < 0.01) at all censoring times of follow-up. The resulting model is reported in Table 4. After adjustment for sex and age at diagnosis, the mortality excess was mainly related to C-AD, G-AD and APS-1 compared to other etiologies. C-AD, G-AD and APS-1 showed similar survival at all censoring time points.

**Table 4:** Paired comparisons at different time points with Cox regression model adjusted for sex and age at diagnosis (aHR = adjusted Hazard Ratio; CI = confidence interval). Adjusted p-values (Bonferroni) are reported

Considered etiology	Reference	Adjusted Cox model								
		Uncensored			Up to 50 years			up to 40 years		
		aHR	95% CI	*p*	aHR	95% CI	*p*	aHR	95% CI	*p*
APS-2/APS-4/I-AAD	G-AD	0.18	0.08–0.40	**< 0.01**	0.17	0.08–0.38	**< 0.01**	0.12	0.05–0.28	**< 0.01**
BA-AD	G-AD	0.22	0.08–0.65	0.12	0.21	0.07–0.62	0.09	0.16	0.06–0.48	**0.02**
ND-AD	G-AD	0.29	0.12–0.72	0.16	0.28	0.11–0.70	0.13	0.22	0.09–0.57	**0.03**
APS-1	G-AD	1.68	0.77–3.66	1.00	1.55	0.7 0- 3.40	1.00	1.17	0.52–2.66	1.00
V/TBC-AD	G-AD	0.26	0.11–0.62	**0.05**	0.24	0.10–0.57	**0.03**	0.17	0.07–0.43	**< 0.01**
C-AD	G-AD	1.99	0.80–4.96	1.00	1.89	0.75–4.71	1.00	1.39	0.55–3.51	1.00
BA-AD	APS-2/APS-4/I-AAD	1.23	0.56–2.70	1.00	1.23	0.56–2.71	1.00	1.33	0.60–2.94	1.00
ND-AD	APS-2/APS-4/I-AAD	1.60	0.92–2.78	1.00	1.64	0.94–2.84	1.00	1.82	1.02–3.22	0.87
APS-1	APS-2/APS-4/I-AAD	9.20	5.75–14.72	**< 0.01**	8.96	5.57–14.43	**< 0.01**	9.51	5.61–16.11	**< 0.01**
V/TBC-AD	APS-2/APS-4/I-AAD	1.43	0.91–2.24	1.00	1.39	0.87–2.20	1.00	1.41	0.86–2.30	1.00
C-AD	APS-2/APS-4/I-AAD	10.94	6.66–17,96	**< 0.01**	10.96	6.65–17.93	**< 0.01**	11.25	6.80–18.62	**< 0.01**
ND-AD	BA-AD	1.30	0.54–3.16	1.00	1.33	0.55–3.22	1.00	1.36	0.56–3.33	1.00
APS-1	BA-AD	7.48	3.21–17.44	**< 0.01**	0.28	3.11–17.01	**< 0.01**	7.15	2.99–17.08	**< 0.01**
V/TBC-AD	BA-AD	1.16	0.51–2.66	1.00	1.13	0.49–2.59	1.00	1.06	0.46–2.46	1.00
C-AD	BA-AD	8.90	3.86–20.54	**< 0.01**	8.87	3.84–20.45	**< 0.01**	8.46	3.67–19.50	**< 0.01**
APS-1	ND-AD	5.74	3.06–10.78	**< 0.01**	5.48	2.91–10.32	**< 0.01**	5.24	2.66–10.30	**< 0.01**
V/TBC-AD	ND-AD	0.89	0.49–1.61	1.00	0.85	0.47–1.54	1.00	0.77	0.41–1.45	1.00
C-AD	ND-AD	6.83	3.68–12.66	**< 0.01**	6.68	3.60–12.37	**< 0.01**	6.20	3.32–11.57	**< 0.01**
V/TBC-AD	APS-1	0.16	0.09–0.27	**< 0.01**	0.15	0.09–0.27	**< 0.01**	0.15	0.08–0.28	**< 0.01**
C-AD	APS-1	1.19	0.64–2.20	1.00	1.22	0.66–2.26	1.00	1.18	0.62–2.25	1.00
C-AD	V/TBC-AD	7.65	4.48–13.07	**< 0.01**	7.88	4.58–13.55	**< 0.01**	8.00	4.59–13.97	**< 0.01**
Considered etiology	Reference	Adjusted Cox model								
		up to 30 years			up to 20 years			up to 10 years		
		aHR	95% CI	p	aHR	95% CI	p	aHR	95% CI	p
APS-2/APS-4/I-AAD	G-AD	0.07	0.03 - 0.16	**<0.01**	0.06	0.02 - 0.16	**<0.01**	0.07	0.02 - 0.22	**<0.01**
BA-AD	G-AD	0.13	0.04 - 0.38	**<0.01**	0.09	0.02 - 0.33	**<0.01**	0.06	0.01 - 0.56	0.29
ND-AD	G-AD	0.17	0.07 - 0.45	**<0.01**	0.20	0.07 -0.57	**0.05**	0.43	0.12 - 1.52	1.00
APS-1	G-AD	0.88	0.37 - 2.06	1.00	0.65	0.24 - 1.79	1.00	0.98	0.30 - 3.19	1.00
V/TBC-AD	G-AD	0.10	0.04 - 0.26	**<0.01**	0.09	0.03 - 0.28	**<0.01**	0.11	0.02 - 0.56	0.15
C-AD	G-AD	0.95	0.37 - 2.42	1.00	0.96	0.35 - 2.64	1.00	2.22	0.66 - 7.47	1.00
BA-AD	APS-2/APS-4/I-AAD	1.85	0.82 - 4.18	1.00	1.42	0.49 - 4.11	1.00	0.91	0.11 - 7.17	1.00
ND-AD	APS-2/APS-4/I-AAD	2.48	1.32 - 4.67	0.11	3.19	1.57 - 6.48	**0.03**	6.50	2.55 - 16.56	**<0.01**
APS-1	APS-2/APS-4/I-AAD	12.58	6.86 - 23.09	**<0.01**	10.56	4.92 - 22.64	**<0.01**	14.85	5.35 - 41.24	**<0.01**
V/TBC-AD	APS-2/APS-4/I-AAD	1.45	0.80 - 2.62	1.00	1.52	0.73 - 3.17	1.00	1.74	0.46 - 6.52	1.00
C-AD	APS-2/APS-4/I-AAD	13.65	8.02 - 23.24	**<0.01**	15.52	8.68 - 27.73	**<0.01**	33.70	15.45 - 73.51	**<0.01**
ND-AD	BA-AD	1.34	0.54 - 3.34	1.00	2.25	0.72 - 6.99	1.00	7.17	0.91 - 56.71	1.00
APS-1	BA-AD	6.81	2.76 - 16.81	**<0.01**	7.44	2.28 - 24.24	**0.02**	16.40	1.96 - 137.26	**0.02**
V/TBC-AD	BA-AD	0.78	0.32 - 1.90	1.00	1.07	0.34 - 3.39	1.00	1.92	0.20 - 18.52	1.00
C-AD	BA-AD	7.39	3.22 - 16.96	**<0.01**	10.94	3.84 - 31.18	**<0.01**	37.21	5.06 - 273.57	**<0.01**
APS-1	ND-AD	5.08	2.42 - 10.63	**<0.01**	3.31	1.39 - 7.89	**0.02**	2.29	0.80 - 6.54	1.00
V/TBC-AD	ND-AD	0.58	0.29 - 1.18	1.00	0.48	0.21 - 1.09	1.00	0.27	0.07 - 0.99	1.00
C-AD	ND-AD	5.51	2.90 - 10.46	**<0.01**	4.86	2.5 - 9.48	**<0.01**	5.19	2.48 - 10.87	**<0.01**
V/TBC-AD	APS-1	0.11	0.06 - 0.24	**<0.01**	0.14	0.06 - 0.35	**<0.01**	0.12	0.03 - 0.48	0.06
C-AD	APS-1	1.09	0.55 - 2.13	1.00	1.47	0.67 - 1.42	1.00	2.27	0.87 - 5.89	1.00
C-AD	V/TBC-AD	9.45	5.12 - 17.44	**<0.01**	10.23	5.04 - 20.75	**<0.01**	19.38	5.95 - 63.10	**<0.01**
At all time points: sex (p = n.s.), age at diagnosis (p < 0.01)										

APS-1, type 1 autoimmune polyendocrine syndrome; APS-2/APS-4/I-AAD, type 2 autoimmune polyendocrine syndrome/type 4 autoimmune polyendocrine syndrome/isolated autoimmune Addison’s disease, BA-AD, bilateral adrenalectomy-related Addison’s disease; C-AD, cancer-related Addison’s disease; G-AD, genetic-related Addison’s disease, ND-AD, not defined Addison’s disease; V/TBC-AD, vascular- or post-tuberculosis-related Addison’s disease

**Fig. 4:**
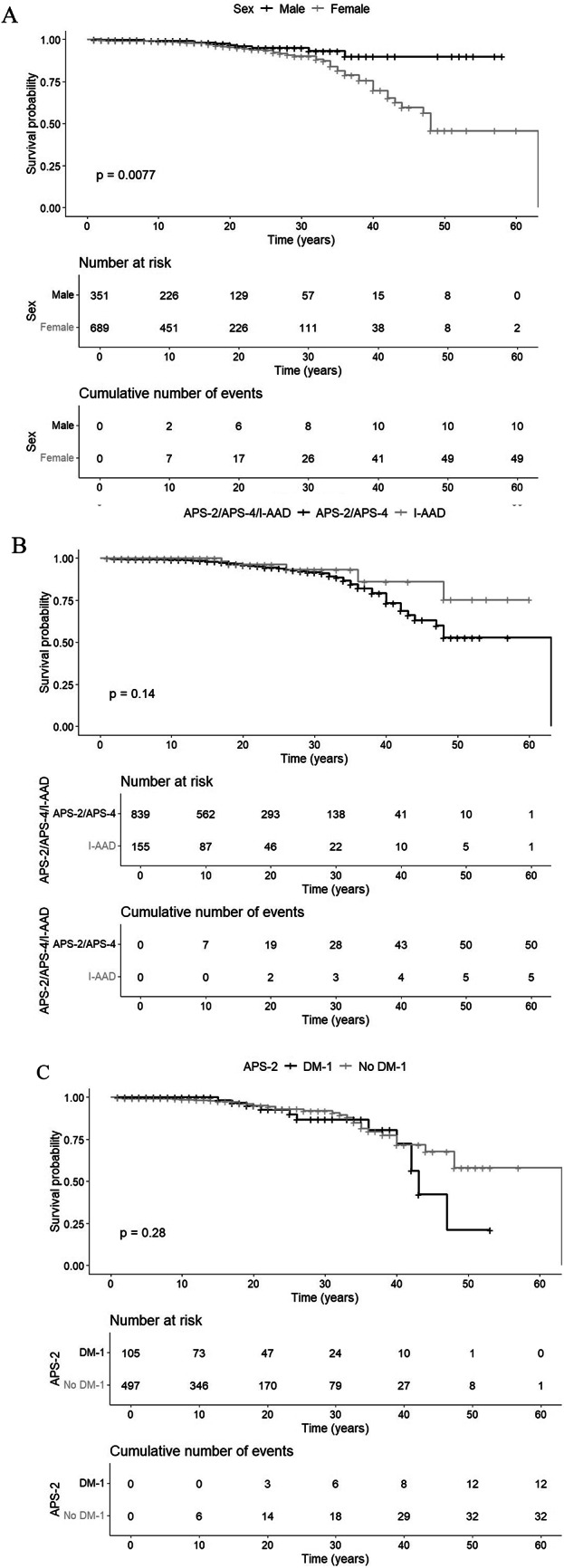
Kaplan–Meier survival curves for: A) APS-2/APS-4/I-AAD based on gender; B) APS-2/APS-4/I-AAD based on isolated (I-AAD) or polyendocrine involvement (APS-2/APS-4); C) APS-2 based on presence or absence of DM-1. APS-2, type 2 autoimmune polyendocrine syndrome; APS-4, type 4 autoimmune polyendocrine syndrome; DM-1, type 1 diabetes mellitus; I-AAD, isolated autoimmune Addison’s disease

Regarding gender-related survival within each etiological group, females in the APS-2/APS-4/I-AAD group had significantly lower survival than males (*p* < 0.01) (Fig. 4 A); this difference emerge on the long term (not significant when censoring at 10, 20 or 30 years of follow-up). Notably, males in G-AD group presented consistently lower survival than females (*p* < 0.01) at all censoring times of follow-up. In the remaining forms, no significant survival difference was observed between males and females: APS-1 (*p* = 0.54), V/TBC-AD (*p* = 0.15), BA-AD (*p* = 0.82), C-AD (*p* = 0.95), and ND-AD (*p* = 0.67) – findings confirmed at various censoring times.

We compared survival curves of patients with APS-2/APS-4/I-AAD to evaluate potential differences between patients with APS-2/APS-4 and those with I-AAD (Fig. 4B). The analysis revealed no statistically significant difference in survival between the two groups (*p* = 0.14), confirmed at various censoring times and in both genders.

A further analysis compared Kaplan–Meier survival curves of patients with APS-2 who have type 1 DM (DM-1) against those with APS-2 without DM-1 (Fig. 4C). The log-rank test revealed no statistically significant difference in survival between the two groups (*p* = 0.28), confirmed at various censoring times and in both genders.

### Standardized mortality ratio

**Fig. 5:**
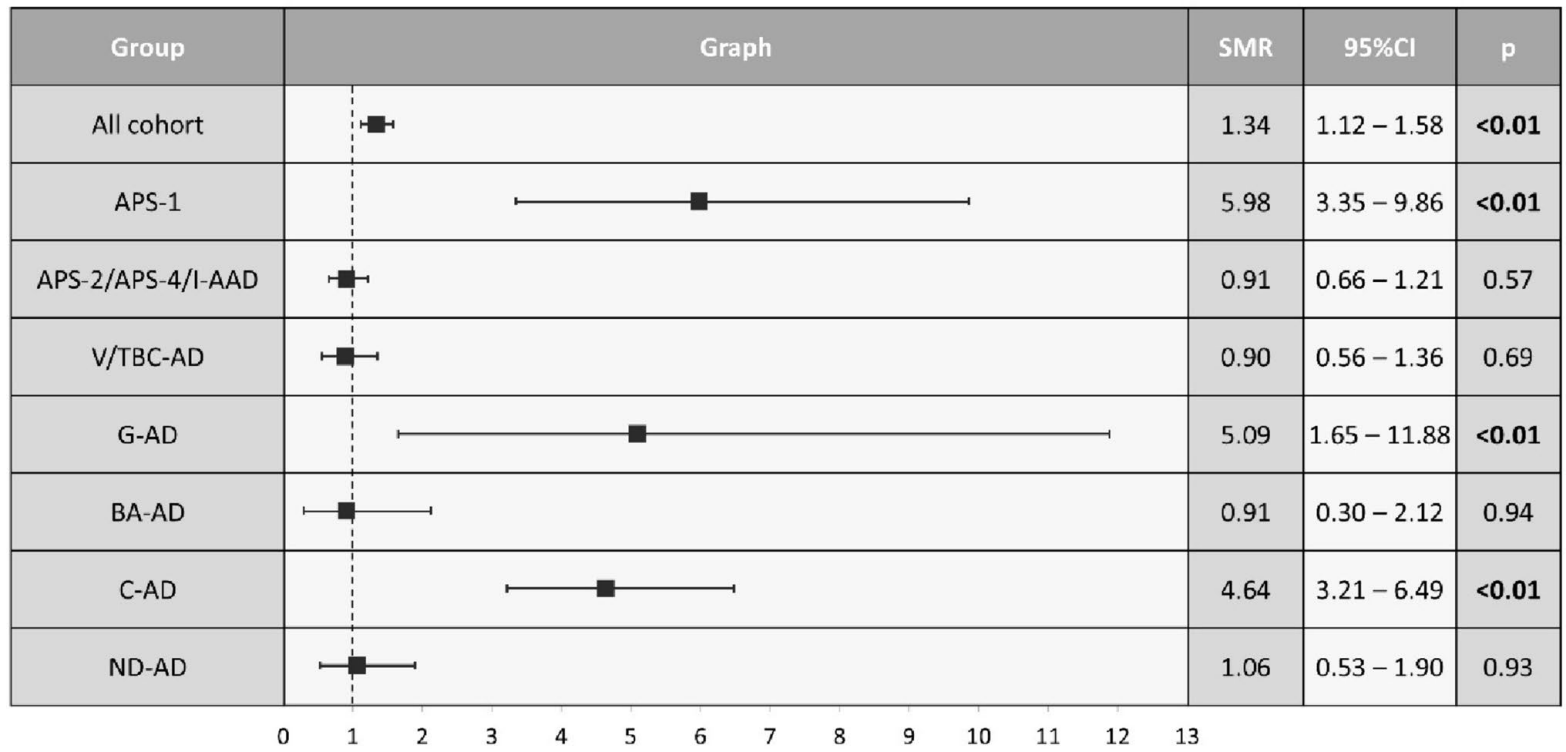
Standardized mortality ratios (SMR) of the study sample and of the different etiological categories of AD during the follow-up between 2011 and 2022 compared to the general population. (CI: Confidence Interval). For etiologies abbreviations see methods section. APS-1, type 1 autoimmune polyendocrine syndrome; APS-2/APS-4/I-AAD, type 2 autoimmune polyendocrine syndrome/type 4 autoimmune polyendocrine syndrome/isolated autoimmune Addison’s disease, BA-AD, bilateral adrenalectomy-related Addison’s disease; C-AD, cancer-related Addison’s disease; G-AD, genetic-related Addison’s disease, ND-AD, not defined Addison’s disease; V/TBC-AD, vascular- or post-tuberculosis-related Addison’s disease

Due to the limited availability of death rates in the general population from official database, the sample for the SMR analysis was reduced to a population of 1,575 patients, including 1,039 (66%) autoimmune cases and 536 patients with other forms of AD (34%) – proportions similar to the whole cohort. During the 2011–2022 period, 138 deaths were registered. The SMR analysis highlighted a significantly increased mortality of the entire sample of patients with AD compared to the general population (SMR 1.34, *p* < 0.01). However, considering the different etiologies of AD, the patients with APS-2/APS-4/I-AAD, V/TBC-AD, BA-AD and ND-AD including 1,188 cases (75.4%) presented a mortality rate comparable to that of the general reference population. On the contrary, an increased mortality was demonstrated in patients with APS-1 (SMR 5.98, *p* < 0.01), with G-AD (SMR 5.09, *p* < 0.01), and with C-AD (4.64, *p* < 0.01), accounting for 387 patients (24.5%) (Fig. 5).

### Survival trends over time

We evaluated survival differences by the date of diagnosis in three groups: (A) diagnosed before 1990 (*n* = 391 cases); (B) diagnosed between 1990 and 2005 (*n* = 680); (C) diagnosed after 2005 (*n* = 755). We performed Cox regression model assessing 10-year survival rate adjusted for sex, age and etiology. Both etiology and age were significant predictors of mortality (*p* < 0.001). Compared to patients diagnosed prior to 1990, the most recent cases presented increased mortality (group B aHR 2.26, 95%CI [0.77; 6.63], *p* = 0.28; group C aHR 3.88, 95%CI [1.37; 11.00], *p* = 0.02). Recent cases were older and presented a reduced prevalence of etiologies associated with the higher mortality (i.e., G-AD, APS-1 AD, C-AD) being 30.7%, 24.7% and 22.0% in group A, B, and C respectively.

## Discussion

Survival and mortality of patients affected by AD has been the subject of debate in literature. Several studies have investigated this crucial aspect, however sample sizes and follow-up durations were very different; moreover, some had only evaluated the hospitalized patients, and most studies also lacked data relating to the impact of the different etiological forms on overall patient survival, as summarized in detail in the introduction (Table 1). Even a recent meta-analysis [14] revised data collected in 7 studies including 9,876 patients with AD, mainly due to autoimmune disease. It confirmed an increased mortality in patients with AD, especially for cardiovascular disease, malignancies, and infections, while among patients with CAH the most common cause of death was adrenal crisis. However, these results require cautious interpretation due to the lack of verification of diagnoses in some studies and to the limited generalizability of these findings mainly from Scandinavian countries and the United Kingdom.

In contrast to previous studies, this study considered a large representative sample of the adult Italian population with primary AD, which included 1,826 patients. For the first time, the different etiologic forms of primary AD were included and separately evaluated and compared with the general population, matched by gender and age of onset of AD. Patients were collected from 30 reference Endocrine Centers in 12 different Italian regions. The follow-up period was 77 years, from 1947 to 2024. The total follow-up period was 31,701 years, with a median follow-up of 15 (6–26) years per patient. This is one of the studies with the largest number of patients with AD evaluated in the longest time period of follow-up to date. Furthermore, for the first time, the sample has tried to represent in its composition the different etiological forms based on their real frequencies present in an adult population. In fact, the sample included patients with AAD and other forms of AD. Patients with AAD (which represented 64.3% of the total sample) were divided in two main categories: APS-1 and APS-2/APS-4/I-AAD. The other forms of AD (which represented 35.7% of the total sample) were divided into V/TBC-AD, G-AD, C-AD, BA-AD and ND-AD forms. Analysis of the groups and subgroups revealed heterogeneity in the median age of onset of AD. In fact, the median ages of onset of adrenal insufficiency in AAD ranged from 10 years in patients with APS-1, to 37 years in patients with APS-2/APS-4/I-AAD, while in patients with other forms of AD the median age varied from 0 years (i.e., diagnosis in the first year after birth, information on the precise month was not available) for G-AD, to 43 years for BA-AD, to 52 years for V/TBC-AD and to 61 years for C-AD. Furthermore, the female to male ratio also varied in the various populations examined, being F > M in APS-1 and APS-2/APS-4 forms, M > F in V/TBC-AD and in I-AAD forms, M = F in the other forms.

Among cases with known cause of death, the leading cause was neoplastic cachexia (63, 30.9%). Apart from C-AD group, death secondary to malignancy was frequent in the APS-1 group (Table 3). On top of oral cancer secondary to chronic candidiasis as previously reported [19], other malignancies associated to APS-1 were gastric and esophageal adenocarcinoma, Kaposi’s sarcoma, colon cancer and peritoneal carcinomatosis. Other causes of mortality in the APS-1 group included: renal insufficiency, fulminant hepatis, adrenal crisis, and diffuse candidiasis.

In line with prior studies [14, 20], we found a significant increased mortality for cardiovascular disease (28, 13.7%) in our cohort. There may be many reasons for this finding, including unphysiological glucocorticoid exposure, chronic inflammation, comorbidities, and aging. Physicians should optimize glucocorticoid replacement by avoiding overtreatment, especially if it is a consequence of a suboptimal mineralocorticoid replacement [21], and mimicking the normal circadian exposure [22]. Novel therapies, such as modified release hydrocortisone formulations, may be useful for these aims. Moreover, healthy lifestyle and correct management of associated comorbidities should be pursued.

The apparently low number of adrenal crises in our cohort should be interpreted cautiously: non-lethal events were not considered in our cohort, the cause of death was unavailable in 36.8% of cases with possible underestimation of the phenomenon, adrenal crises happening prior to the diagnosis were excluded (contrarily to other works on the topic). In addition, patients followed in tertiary level centers are educated to adjust glucocorticoid treatment during intercurrent diseases, possibly reducing the risk of lethal adrenal crises.

The survival analysis, accounting sex and age at diagnosis as covariates, showed a reduced survival of C-AD, APS-1 and G-AD compared to other etiologies, with findings mostly consistent at different censoring points (Table 4). The high mortality of C-AD cases was expected and follows the known aggressive nature of both primary and secondary adrenal malignancies. Both APS-1 and G-AD usually present earlier onset compared to other etiologies, that may prolong the duration of AD and thus justify an increased mortality. Moreover, both present specific comorbidities and complications that may impair survival compared to AD alone. In APS-1 the presence of mutations in the *AIRE* gene induces an important and precocious alteration of the immune system, which on top of AAD leads to many other autoimmune and non-autoimmune diseases, as well as to an immunodeficiency complicated by chronic candida albicans infections. In adrenoleukodystrophy mortality may also be related to neurological comorbidities and complications of the hematopoietic stem cell transplantation (e.g., infections, graft-versus-host disease and graft failure).

SMR analysis allowed comparison of our cohort to a general population matched by gender and age, although our analysis was limited to patients followed during years from 2011 to 2022 due to the availability of mortality rates in the ISTAT database (i.e., official epidemiological data on the Italian population). Data collected in our population showed an increased mortality rate in overall patients with AD compared to the control population. These data are consistent with most previous studies [7, 8, 13, 14]. However, based on the data collected in our study, we observed significant differences in mortality and survival depending on the underlying etiology of AD. Consistently to the prior survival analysis, only C-AD, G-AD and APS-1 presented significantly increased SMR. On the contrary, other etiologies presented normal life expectancy based on the SMR.

Some authors have hypothesized that in patients with AAD the presence of other comorbidities may impact the mortality [23, 24], as discussed above for APS-1 in line with other reports [8, 9, 25]. APS-2/APS-4/I-AAD presented nevertheless preserved life expectancy. To assess a possible role of comorbidities, we compared patients presenting APS-2/APS-4 with those presenting I-AAD alone, but no significant difference in survival was found. Some authors have also demonstrated that the presence of DM-1 increased the mortality among patients with AD [7, 11]. However, when we compared patients with AD with or without DM-1, a similar survival was found.

Regarding the other forms of AD, our study is the first to demonstrate that patients with V/TBC-AD exhibit survival and mortality comparable to the control population, possibly related to a later age of onset of AD. In agreement with previous studies [6, 10, 12], we also demonstrated a high mortality risk in patients with G-AD. We observed a normal survival and mortality in patients with BA-AD, something never defined before.

During these 77 years of follow-up, unexpectedly, the cases diagnosed more recently presented a higher mortality compared with those diagnosed before 2005. This finding may depend on the older age at diagnosis of the recent cases and to a likely “survivor bias”. Indeed, etiologies related to the highest mortality (i.e., G-AD, APS-1 AD, C-AD) were more prevalent among cases diagnosed before 1990, suggesting that only long-surviving cases were included in the study due to its retrospective nature. Therefore, whether an amelioration in the management of these patients over time could have led to mortality reduction in recent years remains unclear.

This study has some limitations, starting with its retrospective design. Notably, the cause of death remained undetermined in 36.8% of cases, with possible underestimation of some entities, including lethal adrenal crises. Patients presented different age at diagnosis and follow-up durations based on etiology. The G-AD group comprised different causes, possibly influencing the results and preventing more precise evaluation of each genetic disease. Despite most findings presenting concordant trends in the sensitivity analyses of survival curves, due to the long range of time considered a selection bias towards long-surviving cases is likely. Moreover, SMR results are affected by the restriction of the analysis to recent years: due to limited availability of data from the Italian general population, the initial sample was reduced to a population of 1,575 patients presenting 138 deaths. Thus, further studies aiming to a comprehensive SMR estimate are needed to confirm our findings. Some potentially important confounding variables were also not available (e.g., smoking habit, other comorbidities). Nevertheless, the large sample size included in this study may help mitigate the impact of these limitations and reduce potential bias in our findings.

In conclusion, our study demonstrates substantial variation in age at onset, female-to-male ratio, mortality and survival among adult patients with AD, according to the underlying etiological subtype. Therefore, we suggest that in the future survival and mortality should be analysed separately for each AD subtype comparing the data with age- and sex-matched control groups.

## Data Availability

All data generated or analysed during this study are included in this published article.

## Declarations

U.P. is the Editor-in-Chief of the Journal of Endocrinological Investigation and played no role in the Journal’s evaluation of the manuscript. C.Si. received occasional consulting honoraria from BresMed, Neurocrine, Recordati and Clearview Healthcare Partners LLC. A.S. received grant support from Recordati Rare Disease, and Esteve.
